# Development, Validation and Application of an Ultra-High-Performance Liquid Chromatography–Tandem Mass Spectrometry (UHPLC-MS/MS) Method after QuEChERS Cleanup for Selected Dichloroanilines and Phthalates in Rice Samples

**DOI:** 10.3390/foods11101482

**Published:** 2022-05-19

**Authors:** Emmanouil Tsochatzis, Olga Begou, Stavros Kalogiannis, Helen Gika, Emel Oz, Fatih Oz, Georgios Theodoridis

**Affiliations:** 1 Department of Food Science, Aarhus University, Agro Food Park 48, 8200 Aarhus, Denmark; emel.oz@atauni.edu.tr (E.O.); fatihoz@atauni.edu.tr (F.O.); 2 Laboratory of Analytical Chemistry, Department of Chemistry, Aristotle University of Thessaloniki, University Campus, 54124 Thessaloniki, Greece; mpegolga@chem.auth.gr (O.B.); gtheodor@chem.auth.gr (G.T.); 3 Biomic_AUTh, Center for Interdisciplinary Research and Innovation (CIRI-AUTH), Balkan Center, B1.4, 10th km Thessaloniki-Thermi Rd, 57001 Thessaloniki, Greece; kalogian@ihu.gr (S.K.); gkikae@auth.gr (H.G.); 4 Department of Nutritional Sciences and Dietetics, International Hellenic University, Sindos, 57400 Thessaloniki, Greece; 5 Laboratory of Forensic Medicine and Toxicology, School of Medicine, Aristotle University of Thessaloniki, University Campus, 54124 Thessaloniki, Greece; 6 Agriculture Faculty, Food Engineering Department, Ataturk University, Erzurum 25240, Turkey

**Keywords:** phthalates, dichloroanilines, rice, QuEChERS cleanup, UHPLC-MS/MS analysis

## Abstract

Dichloroanilines and phthalic acid esters (phthalates) are food contaminants, stable in solution even at high temperatures, which exhibit considerable toxic effects, while acting as endocrine disruptors. In the present study, a quick and easy UHPLC-MS/MS method for simultaneously analyzing two dichloroanilines (3,4-DCA and 3,5-DCA) and six phthalates (DMP, DnBP, BBP, DnOP, DEHP, and mBP) in commercial rice samples was developed, validated, and applied. For the cleanup process, the methodology of quick, easy, cheap, effective, rugged, and safe (QuEChERS) was applied, whereas different dispersants (GCB, C18, and PSA) were tested. What was developed and presented had limits of detection ranging from 0.017 up to 0.12 mg/kg, recoveries (trueness) below 120%, and relative standard deviations (RSD; precision) <15% for all target analytes, whilst no significant matrix effects occurred for all analytes. It was determined that the rice samples analyzed using this developed technique did not contain any of the two dichloroaniline compounds (3,4-DCA and 3,5-DCA) nor two of the six phthalate (DMP and mBP) compounds analyzed, while the levels of other phthalates (DEHP, BBP, DnBP and DnOP) were within the legal limits. The current method ensures a fast and easy approach for the high-throughput quantification of the selected food contaminants in rice.

## 1. Introduction

Rice is the grain product of the paddy, which is the grain of the cultivated plants of the *Oryza sativa* L. species, obtained by removing the embryo, husk, and aleuron partially or completely, by applying various milling processes after being peeled, in accordance with the technique. It is known that rice has been consumed for many years and feeds about half of the population [1], while it is known to be the most-produced grain after wheat and corn. Rice is grown in more than 100 countries, and on every continent. On the other hand, agricultural products can only be produced in the desired quantity and quality, provided that they are protected from disease factors and pests. In this context, the use of pesticides comes to the fore. Pesticides are widely used for the destruction, removal, and reduction of pests that may cause negative effects during the production, transportation, and storage of various agricultural products [2]. However, the use of pesticides at high levels, during the production of cereals such as rice, might impact the quality of the final product and lead to higher residues amounts [3,4]. One of the most important pesticide residue groups is dichloroanilines (DCAs). DCAs, primarily 3,4-dichloroaniline (3,4-DCA) and 3,5-dichloroaniline (3,5-DCA), have been characterized as Endocrine Disrupting Chemicals (EDCs), due to their high toxicity levels and because they end up in foods as a result of pesticide degradation. In particular, 3,4-DCA is known to be the main metabolic product of propanil, a widely used pesticide in rice cultivation [3,4,5], while 3,5-DCA is known to result from the dicarboximide fungicides vinclozolin and iprodione, which are used in agriculture [6].

Phthalic acid esters (phthalates PAEs) are commonly used in industrial, agricultural, and domestic applications, mainly as plasticizers [7]. There are two main categories of phthalates, namely low- and high-molecular-weight compounds, and they can be found in a plethora of food matrices, during manufacture, use, or disposal, due to their migration capacity. Human exposure to phthalates is almost unavoidable, due to their extensive use stability in solutions and resistance to high temperature [8]. The main sources of human exposure to phthalates are known to be air, water, cosmetics, pharmaceutical products, and diet [9,10,11,12,13,14,15]. Water sources, such as rivers and seawater, which are widely used in paddy fields, are important sources of contamination for phthalates [3,4]. Due to the fact that phthalates negatively affect the human endocrine system and hormone production, they are characterized as EDCs and Hormonally Active Agents (HAAs). Studies reported the occurrence of effects on the human reproductive systems, in relation to exposure to phthalates. Thus, it is reported that the immature male reproductive system is the most sensitive system, and phthalate exposure causes an increase in the incidence of undescended testicles, a decrease in testicular weights, and a decrease in the anogenital distance [9,16]. Studies have, also, shown that phthalates are related to fertility and growth problems, allergies, asthma, and changes in cord-blood hormone levels [17,18,19]. Due to their negative effects on health, various authorities have imposed restrictions for phthalates. Tolerable daily intake (TDI) values have been set as 50 μg/kg body weight per day (expressed as DEHP equivalents), by the European Food Safety Authority (EFSA) and the World Health Organization (WHO) [20]. Thus, their determination in foods is of great significance.

**Figure 1:**
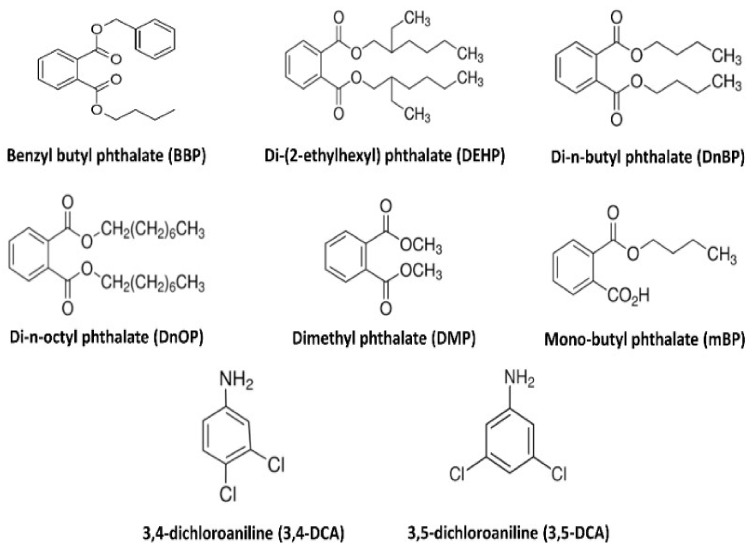
Chemical structures of the studied food pollutants.

For the analysis of dichloroanilines and phthalates, techniques such as liquid chromatography, gas chromatography, thin layer chromatography (TLC), fluorescence measurement, ultraviolet (UV) spectrometry, direct analysis in real time, micellar electrokinetic chromatography, and diode array detection (DAD) have been reported in the literature [7]. However, in recent years, more advanced techniques have been employed, such as capillary electrophoresis (CE) [21,22], liquid chromatography (LC) [9,23], or gas chromatography (GC) coupled with mass spectrometry (MS) [9,24,25], due to their satisfactory resolution for all aforementioned analytes. Due to the fact that the matrix complexity seen in agricultural products can affect the analysis sensitivity of the target contaminants, it is very important to apply appropriate sample pretreatment techniques to eliminate matrix interferences. The most common cleanup and extraction pretreatment techniques are solid-phase extraction (SPE), solid-phase microextraction (SPME) [4,7], liquid–liquid extraction (LLE) [24,25], solid-phase extraction (SPE) [26], matrix solid-phase dispersion (MSPD) [3,4] and solid-phase micro-extraction [14,19]. The use of acetonitrile or ethyl acetate presents sufficient extraction recovery and satisfactory matrix cleanup. Other studies have employed sorbent-based extraction methodologies; dispersive solid phase extraction is a pretreatment technique, with proven efficiency in the extraction of a large spectrum of pesticides from foods, including complex plant and animal tissues. Its application is based on blending inorganic solids (e.g., alumina, florisil), using QuEChERS (Quick, Easy, Cheap, Effective, Rugged and Safe) protocols [27], or using modified QuEChERS protocols [23,27]. Recent advances in pesticide analysis involve the use of molecularly imprinted polymers or enantiomeric/chiral chromatography [28,29,30]. However, limited work has been done for the analysis of phthalates and dichloroanilines in dry foods, following a fast and efficient sample preparation such as QuEChERS. The application of simple and quick extraction protocols, in connection to sensitive analytical methodologies, provides a tool for high-throughput analysis. Analytes of interest have been determined in a great deal of foods such as, tea, dairy products, milk, fish and meat products, wine, grape spirits, oily products, vegetables and fruits, cereal products, water, and soft and sport drinks [7,15,23,31,32,33,34,35]. The European Reference Laboratory for Food Contact Materials (EURL-FCM) showed that GC-MS and LC-MS/MS are the predominant techniques of choice for the official control of these analytes [36]. In addition, it was highlighted that these compounds and, especially, three regulated phthalates [37], namely diallyl phthalate (DAP; FCM316), dibutyl phthalate (DnBP; FCM157), and benzyl butyl phthalate (BBP; FCM 159), proved to be unstable in a solution system containing 10% *v*/*v* ethanol, while the content of DEHP was shown to be adequately homogeneous and stable [36]. The major difficulties regarding phthalates analysis are, on the one hand, the complexity of food matrices that may induce significant matrix effect or interferences, and, on the other hand, the risk of contamination by plastic equipment and parts of the instrumentation at almost all analytical steps, from sampling to final analysis. Furthermore, preventing hydrolysis of these diesters has to be taken into consideration, prior to analysis. Different methodologies have been adopted to minimize such problems, including use of high purity solvents, glassware cleaning with oxidizing reagents, water, and organic solvents, and/or calcination in high temperatures. Therefore, in the present study, it was aimed to develop and validate a fast and sensitive ultra-high-performance reversed-phase liquid chromatography–tandem mass spectrometry (UHPLC-MS/MS) method for two dichloroanilines and six phthalates compounds (Figure 1) and to, simultaneously, determine these compounds in ground rice samples with a validated QuEChERS extraction process.

## 2. Materials and Methods

### 2.1. Chemicals and Materials

LC-MS-grade acetonitrile (MeCN) and H_2_O were purchased from Fluka (Steinheim, Germany). A Milli-Q purification device (Millipore Direct-QR 3, Millipore International, Merck KGaA, Darmstadt, Germany) was used for obtaining the water used in the analyses. Acetone (>99%) and hexane (HPLC grade) were obtained from Centralchem (Bratislava, Slovakia) and VWR Chemicals (Radnor, PA, USA), respectively. Magnesium sulphate (MgSO_4_), graphitized carbon black (GCB), C18 sorbents, and primary secondary amine (PSA) were supplied by Agilent Technologies (Santa Clara, CA, USA). The aniline standards, namely 3,4-dichloroaniline (3,4-DCA) and 3,5-dichloroaniline (3,5-DCA), were obtained from Riedel de Haën (Seelze, Germany), with purity higher than 99.9%. Phthalate analytical standards, such as dimethyl phthalate (DMP), di-n-butyl phthalate (DnBP), mono-butyl phthalate (mBP), benzyl butyl phthalate (BBP), di-n-octyl phthalate (DnOP), and di-(2-ethyl hexyl) phthalate (DEHP)), were supplied by Sigma Aldrich (Steinheim, Germany).

### 2.2. Samples

Ten samples of commercial rice in polymeric bags were purchased from the local market in Thessaloniki, northern Greece. The rice products purchased were used as a sample, after they were ground in a Retzch mill (model ZM 1000, 0.075 mm sieve). All mill parts were cleaned and rinsed with acetone and hexane, and a ground sample was collected only from the part that was not in contact with the metallic reservoir, in order to eliminate potential contamination by this machinery.

### 2.3. Preparation of Standard Solutions, Calibration Standards and Quality Control Samples (QCs)

For every food contaminant studied in the present study, stock solutions (1000 μg/mL) were prepared by using MeCN and storing at −18 °C for one month. Working standards were prepared by diluting the stock solutions, stored at 4 °C and used for up to one week.

Quality Control samples (QCs) were prepared at three concentration levels, including low (LQC), medium (MQC), and high (HQC) concentrations for dichloroanilines (0.5 mg/kg, 2 mg/kg, and 10 mg/kg) and phthalates (0.05 mg/kg, 0.5 mg/kg, and 2 mg/kg).

### 2.4. UHPLC-MS/MS Analysis

The selected food contaminants were determined by using a UHPLC-MS/MS system, an Accela TSQ Quantum Access MAX Triple Quadrupole Mass Spectrometer system with XCalibur v2.0 software (Thermo Scientific, San Jose, CA, USA). While the tray temperature of the autosampler was set at 10 °C, the separation process was performed at 30 °C at a flow rate of 200 μL/min on a reversed phase analytical column (U-VD SPHER Pure TUR 100 C18-E 1.8 μm, 100 mm × 2.0 mm column equipped with a 5.0 mm × 2.0 mm C18 guard cartridge), using solvent A (MeCN containing 0.1% formic acid) and solvent B (H_2_O containing 0.1% formic acid) solvents. The gradient program was as follows: from 40% A to 60% A (0–6 min), subsequently to 100% A (6–8 min), followed by an isocratic elution from 8–14 min. A system equilibration to 40% A was performed (14–17 min). The total analysis time was 17 min, and a 5 μL injection was made from the extracts. After every injection cycle, the injection needle was washed with a strong organic solvent (MeCN:MeOH:H_2_O, 60:30:10, *v*/*v*/*v*) in order to prevent carryover or cross-contamination. Furthermore, both solvent and procedural blanks were injected at the beginning of analyses, in order to evaluate possible contamination originating from the solvents, materials, and analytical system.

The Selected Reaction Monitoring (SRM), in its electrospray positive ionization mode (ESI+), was used for the detection of these food pollutants. Source parameters were set as follows: spray voltage: 3000 V; capillary temperature, 300 °C; vaporizer temperature, 350 °C; sheath gas pressure, 35 arbitrary units (Arb); aux gas pressure, 10 Arb; ion sweep gas pressure at 2.0 Arb; and ion source discharge current, 4.0 μA, while argon was used as the collision gas at a pressure of 1.5 mTorr. Tube lens voltage and collision energy were manually optimized for each of the studied analytes, separately, by direct infusion into the mass spectrometer (Table 1).

### 2.5. Sample Preparation

For the efficient extraction of all analytes, different dispersive solid phase extraction (d-SPE) or QuEChERS cleanup [27] was applied and assessed in terms of extraction efficiency. After extracting the ground rice with MeCN in the presence of MgSO_4_, different sorbents or sorbent mixtures were applied. The sorbents tested were C18, PSA, and combinations of these two treatments with GCB, in the presence of 75 mg/mL of anhydrous MgSO_4_.

First, 10 mL of MeCN and 4 g MgSO_4_ were put into a precleaned borosilicate glass tube, and 10 g of the ground rice sample was added into this tube. Solvents were measured using automatic pipettes with pre-rinsed glass tips (single use). After each tube was tightly capped and intensively vortex mixed for 1 min, the tube was centrifuged for 5 min at 1780× *g* at 4 °C. A 2 mL aliquot of the clear supernatant was transferred to a glass vial supplied with an aluminum screwcap, in which anhydrous MgSO_4_ (150 mg) and the aforementioned sorbents or sorbent mixtures (100 mg) were placed. After the tube was tightly capped and intensively vortex mixed for 1 min, the tube was centrifuged for 5 min at 1780× *g* at 4 °C. Then, the supernatant was collected, condensed to 0.5 mL under nitrogen at 40–45 °C, and filtered by using a PTFE filter (0.22 μm). An aliquot amount of the final extract was transferred into an autosampler vial for UHPLC-MS/MS analysis, and the injection volume was 5 μL. As phthalates are ubiquitous in the environment, all glassware were properly cleaned, rinsed with acetone and hexane, and baked at 400 °C (non-volumetric glassware) to avoid cross-contamination of phthalates from the laboratory environment, following all guidelines [11]. All cleaned glassware were stored in a desiccator containing aluminum oxide (Al_2_O_3_). Moreover, to ensure absence of phthalate traces, a blank extraction was performed, using equipment cleaned prior to extraction.

### 2.6. Method Validation

#### 2.6.1. Linearity, Limit of Detection, and Limit of Quantification Values

The method’s linearity was assessed by analyzing standard solutions mixtures at 7 different concentration levels for all food pollutants studied, representing the working concentration range. By plotting the peak area of analytes, the calibration curve was constructed, and to evaluate linearity (R^2^), a linear regression was performed. Limit of detection (LOD) equal to 3 times the signal-to-noise (S/N) ratio), and limit of quantitation (LOQ) equal to 10 times the S/N ratio, values were calculated [38,39]. Quantification of real samples was performed, according to the external calibration method, at 7 different concentration levels.

#### 2.6.2. Precision and Accuracy

Precision and accuracy were assessed with standard solutions such as intra-day accuracy and inter-day accuracy (for three consecutive days), by evaluating relative standard deviation at three different concentration levels. Both intra-day and inter-day precision and accuracy were also assessed in spiked rice samples, performing the aforementioned experiments at three levels: 0.5 mg/kg (LQC), 2 mg/kg (MQC), and 10 mg/kg (HQC) for dichloroanilines, and 0.05 mg/kg (LQC), 0.5 mg/kg (MQC), and 2 mg/kg (HQC) for phthalates. The analytical method’s accuracy was expressed as relative error, and precision was expressed as relative standard deviation (RSD, %).

#### 2.6.3. Extraction Efficiency

Extraction recovery rates were determined using the standard addition method (by adding at different concentrations of the analytes to rice samples, *n* = 2). The solvent tested for the extraction of the selected analytes was acetonitrile, following previously reported scientific works [23,27]. The samples were fortified to obtain a nominal concentration of 1.0 mg/kg for each analyte and treated as described in detail in Section 2.5, before being subjected to UHPLC-MS/MS analysis. Recovery (RE, %) was expressed as a percentage and calculated by using the follow equation:

(1) $$RE\;\left(\%\right)=100\;\times \;\left(\frac{analyte\;peak\;area\;spiked\;before\;extraction}{analyte\;peak\;area\;spiked\;after\;extraction}\right),$$

#### 2.6.4. Matrix Effect

Once using MS detection, evaluation and assessment of the matrix effect (ME) is important, in order to check potential ion suppression or enhancement that could interfere in the analysis of the target substances. The ME was calculated by using the equation below, based on existing approaches [40,41]:

(2) $$ME\;\left(\%\right)=\left(\frac{B-C}{C}\right),$$

With B being the slope of regression fit of results for the standards added to the samples, after going through the sample preparation steps, and C being the slope of regression fit of results, for target analytes standards in MeCN.

While values higher than 10% indicate ion enhancement, and values lower than −10% indicate ion suppression, values from −10% up to 10% indicate no relevant ME. Matrix effect (%) was studied in one concentration (1 mg/kg) as well as in diluted samples, to assess the possibility of use for reducing or eliminating potential signals suppression [38,40,42].

## 3. Results

### 3.1. Optimization of QuEChERS Cleanup

**Figure 2:**
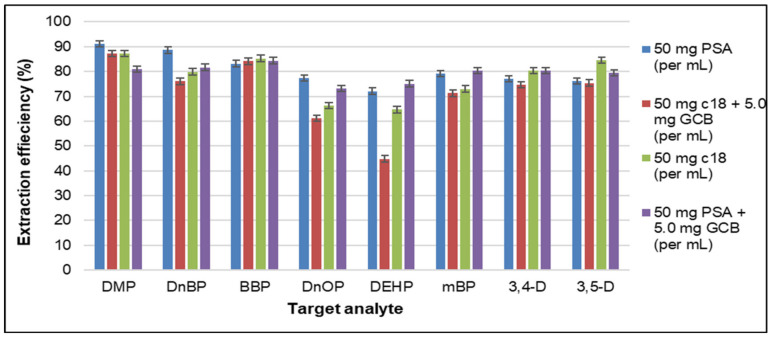
Rice sample QuEChERS cleanup efficiency, using different sorbents in fortified rice samples at the 0.25 mg/kg level.

The optimization of the QuEChERS cleanup was achieved by calculating the extraction efficiency, as described in Section 2.6.3, using different types and amounts of sorbents, namely PSA, GCB, and C18, or sorbent combinations. Comparison of the extraction efficiency of the applied QuEChERS cleanup protocols is illustrated in Figure 2. The addition of salt, and especially MgSO_4_, ensures the absorption of H_2_O residues that might hydrolyze the esters [36], which might increase extraction efficiency via an increase in the ionic strength of the solutions [43]. Moreover, a procedural blank was performed to assess the cross-contamination from glassware, where no amounts of phthalates nor dichloroanilines were identified.

**Figure 3:**
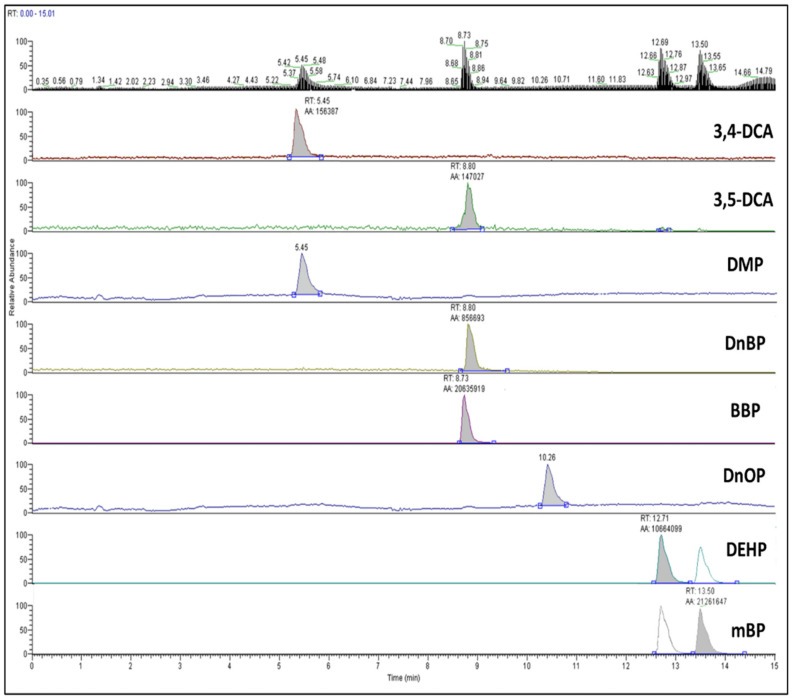
TIC chromatogram and MRM chromatograms in spiked rice samples (1.0 mg/kg for all target analytes), after QuEChERS cleanup.

Based on the obtained results, application of PSA alone was the most efficient, having the highest mass fractions of all analytes. In the case of C18 sorbent (50 mg per mL), a decrease in the isobars DEHP and DnOP was observed, with the efficiency being less than 60% for both analytes. The combination of C18-GCB (50 mg + 5.0 mg per mL) had an even more pronounced effect on these isobars, reducing their extraction efficiency to 55% and 35%, respectively, indicating a strong binding effect between the analytes and the sorbent material. Fifty milligrams of PSA-GCB (50 mg + 5.0 mg per mL) proved to, also, be efficient, presenting the same effect for both DEHP and DnOP, though with higher extraction efficiency (65% and 64%, respectively). Thus, it could be concluded that the best cleanup approach has been achieved by using only PSA at a level of 50 mg per mL extract, as the extraction efficiency was calculated to be above 72% for all target analytes. PSA was selected as the optimum sample preparation protocol. The use and the extraction efficiency of PSA, in the case of phthalates, was, also, reported in different food matrices, as in tea by Yadav et al. [44], in mussels [23], and in various food samples by Cao et al. [45], where ethyl acetate or acetonitrile were preferred as extraction solvents. The chromatograms of spiked rice grain samples, extracted with the validated QuEChERS cleanup protocol, are presented in Figure 3.

Since hydrolysis of the diesters may also affect the analysis, especially at pH values above 7 and below 5, the removal could be beneficial [16,46]. QuEChERS methodology may also reduce the risk of hydrolysis, since inorganic salts used, such as MgSO_4_, which may absorb and, thus, remove residual H_2_O.

### 3.2. Method Validation

#### 3.2.1. Linearity and Limit of Detection (LOD) and Limit of Quantification (LOQ)

Limit of detection (LOD) and limit of quantification (LOQ), the upper linear range, and coefficients of determination (R^2^) of all analytes, are provided in Table 2. Coefficients of determination ranged from 0.999 for BBP and DnOP to 0.9998 for 3,4-DCA and 3,5-DCA. LODs and LOQs were 0.12 mg/kg and 0.35 mg/kg for both dichloroanilines, respectively, while in the case of phthalates, LODs ranged from 0.007 mg/kg (BBP) to 0.017 mg/kg (DMP) and LOQs from 0.020 mg/kg (BBP) to 0.045 mg/kg (DMP).

The results proved to be highly comparable to similar methodologies applied to other solid food matrices [23], although LOD and LOQ values were slightly higher compared to liquid matrices [15].

#### 3.2.2. Precision and Accuracy

The method’s trueness was assessed in standard solutions at three levels and was found to be at acceptable levels, with recoveries ranging from 93% to 105%, for both dichloroanilines and phthalates. Precision expressed in RSDs (%) were in values lower than 15% for all target analytes.

In spiked rice samples, accuracy ranged from 90.3% (3,4-DCA) to 117.8% (DnOP) in the intra-day assay and from 90.0% (DEHP) to 97.8% (BBP) in the inter-day assay. The respective precision, expressed as %RSD, was found to be from 1.3% (mBP) to 14.9% (DEHP) and from 1.2% (DnBPs) to 12.9% (DnOP) for intra- and inter-day results, respectively. All precision and trueness results are presented in Table 3.

Obtained results are in accordance with Yadav et al., who applied a modified QuEChERS using ethyl acetate, instead of acetonitrile. Reported recoveries range from 70% to 101% (for all phthalates), while RSDs (%) were found to be less than 2%. Moreover, the reported LODs and LOQs are highly comparable with the ones reported for other food components, such as tea [44], mussels [23], and alcoholic beverages [15]. In another study from Cao et al., where different phthalates in various food matrices were studied in the framework of a screening survey, applying QuEChERS in cereals presented recoveries ranged from 91.4% to 115% for the respective phthalates and presented RSDs (%) of less than 11% [45].

#### 3.2.3. Quantification and Matrix Effect

Quantitation was performed by the external calibration curve of the analytes. Due to the fact that no matrix effect (ME) was observed, external calibration can be used without inducing errors. Since the matrix effect can be reduced by a sample or sample extract dilution, the samples were diluted, accordingly, to identify the effects of dilution, whilst the calculations were based on the respective diluted mass fractions. The results demonstrated that, in the case of the studied mass fraction level of 1 mg/kg (Table 2), no relevant ME was identified. Furthermore, performed dilutions did not present any additional effects, since ME (%) ranged between −3.2% (BBP) up to +2.9%, as illustrated in Figure S1. The results indicated that there was no signal suppression in all studied cases, as the same level of ME (%) was observed in the different dilutions [38,40,42]. Hence, the dilution approach could be used, if needed, as a potential tool to eliminate or to significantly reduce signal suppression [38,40,42]. Overall, based on the ME% values, it can be concluded that no significant matrix effect is present in the suggested method.

### 3.3. Analysis of Real Samples

Ten real rice samples purchased from local markets of Thessaloniki, Greece were analyzed with the developed and validated method to assess the potential mass fractions of the studied dichloroanilines and phthalates. All results are presented in Table 4.

For all of the dichloroanilines (3,4-DCA and 3,5-DCA) and two phthalates (DMP and mBP) studied in the present study, the mass fractions were found below LOD in all samples analyzed (Table 4). Regarding dichloroanilines (3,4-DCA and 3,5-DCA), the data obtained in the present study show that in Greece, chlorinated anilines are not used during rice production. On the other hand, it is known that chlorinated anilines are degradation products of some pesticides, such as propanil, which is used in rice applications. Therefore, dichloroanilines can be found in rice, in certain cases [5,24]. Indeed, there are studies in the literature emphasizing that pesticides containing dichloroaniline moieties are used extensively in rice production [47]. As for phthalates, the most abundant analyte was DEHP, followed by BBP, DnBP, and DnOP. DEHP was, especially, present in 60% of the samples, although in low concentrations (below 0.30 mg/kg). Similarly, there are other studies in the literature showing that DEHP is the most commonly detected phthalate in rice. As a matter of fact, in a study by Skribic et al. (2012) [48], in which they determined the phthalate levels of rice samples they bought from Serbian and Chinese markets, it was reported that the most common phthalate determined was DEHP. Many phthalates are ubiquitous, especially DEHP, either in the environment [4,9] or due to their extensive use as a regulated plasticizer for food-packaging applications [4,15,25]. Therefore, their presence could be expected. For the latter, especially DEHP, BBP, and DnBP, legislative limits in foods of 1.5, 30, and 0.3 mg/kg exist [37]. On the other hand, there are studies in the literature showing that phthalates are determined in rice at levels higher than the results obtained in the present study [48]. The differences between the results obtained in the current research and the results in the literature could be due to factors such as the rice type, storage conditions and duration, analysis technique, etc.

## 4. Conclusions

The developed UHPLC-MS/MS method was aimed in the simultaneous quantification of two dichloroanilines (3,4-DCA and 3,5-DCA) and six phthalates (DMP, DnBP, BBP, DnOP, DEHP, mBP). The selection of the specific analytes was based on the imperative necessity of their quantification in the consumable food matrices of the everyday diet. The developed and validated method provided a quick sample preparation procedure, low qualitative and quantitative detection limits, good chromatographic separation, accuracy, recovery, precision, and non-significant matrix effect. A QuEChERS (quick, easy, cheap, effective, rugged, safe) method was used and validated for the sample preparation, and PSA proved to be the most effective for ground rice samples cleanup. It was determined that the rice samples analyzed using this developed technique did not contain either of the two dichloroanilines (3,4-DCA and 3,5-DCA) and two of the six phthalate (DMP and mBP) compounds analyzed, while the levels of other phthalates (DEHP, BBP, DnBP, DnOP) were within the legal limits. Thus, it has been proven in the present study that these hazardous chemicals can ben simultaneouslyn analyzed with this developed analysis technique. In addition, the presented method provides an easy and fast approach for the quantification of these selected pollutants in rice.

## Figures and Tables

**Table 1:** Target analytes detection parameters (MRM) and retention times.

Analytes	Molecular Formula	Monoisotopic Mass (Da)	Precursor Ion (*m*/*z*)	Qualifier Ion (*m*/*z*)	Collision Energy (V)	Tube Lens (V)	Ret. Time (Average Min ± S_d_)
3,4-DCA	C_6_H_5_Cl_2_N	160.979	162.046	99.781	38	77	5.64 ± 0.09
3,5-DCA	C_6_H_5_Cl_2_N	160.979	162.102	128.106	34	99	8.83 ± 0.15
Dimethyl phthalate (DMP)	C_10_H_10_O_4_	194.058	195.113	163.200	19	55	5.57 ± 0.13
Di-n-Butyl phthalate (DnBP)	C_16_H_22_O_4_	278.152	279.204	149.100	17	39	8.73 ± 0.09
Benzyl butyl Phthalate (BBP)	C_19_H_20_O_4_	312.136	313.189	91.220	23	42	8.65 ± 0.09
Di-n-octyl phthalate (DnOP)	C_24_H_38_O_4_	390.277	391.398	261.220	11	39	10.30 ± 0.10
Di-(2-ethyl hexyl) phthalate (DEHP)	C_24_H_38_O_4_	390.277	391.406	279.340	16	53	12.74 ± 0.09
Mono-butyl phthalate (mBP)	C_12_H_14_O_4_	222.089	223.150	149.150	19	55	13.44 ± 0.12

**Table 2:** Limits of detection (LODs), limits of quantification (LOQs), linear ranges, and linear regression coefficients for target analytes.

Target Analyte	LOD (mg/kg)	LOQ (mg/kg)	Upper Linear Range (mg/kg)	R^2^	Matrix Effects (%) *
DMP	0.017	0.045	2.0	0.9992	0.1
DnBP	0.008	0.020	2.0	0.9992	0.2
BBP	0.007	0.020	2.0	0.9990	0.3
DnOP	0.014	0.036	2.0	0.9990	0.2
DEHP	0.012	0.035	2.0	0.9991	0.4
mBP	0.008	0.020	2.0	0.9994	1.3
3,4-DCA	0.12	0.35	15	0.9998	1.1
3,5-DCA	0.12	0.35	15	0.9998	0.5

* Values from −10% up to 10% indicate no relevant ME. See also Section 3.2.3 and Figure S1.

**Table 3:** Precision and trueness results for the analysis of target analytes in spiked rice.

Target Analyte	Added (mg/kg)	Intra-Day (*n* = 6)			Inter-Day (*n* = 3 × 3)		
		Calculated Mean Conc. * mg/kg	Recovery (%)	RSD (%)	Calculated Mean Conc. * mg/kg	Recovery (%)	RSD (%)
DMP	0.05	0.048	97.0	5.0	0.044	90.9	1.9
	0.5	0.534	107	4.9	0.491	92.0	7.2
	2	1.840	92.1	11.9	1.789	97.1	1.9
DnBP	0.05	0.049	97.2	6.9	0.047	93.6	8.9
	0.5	0.53	106	5.1	0.51	102	5.9
	2	2.010	101	5.0	1.941	96.4	1.2
BBP	0.05	0.058	115	14.7	0.052	90.3	2.0
	0.5	0.580	115	5.9	0.543	94.1	11.4
	2	2.173	109	6.9	2.125	97.8	2.4
DnOP	0.05	0.046	92.8	12.3	0.043	92.5	8.0
	0.5	0.590	117	8.9	0.535	90.8	12.9
	2	1.950	97.3	2.2	1.766	90.8	3.5
DEHP	0.05	0.052	104	14.9	0.047	90.2	10.5
	0.5	0.542	108	5.6	0.493	91.0	12.8
	2	2.050	102	6.6	1.842	90.0	4.6
mBP	0.05	0.056	113	9.5	0.051	90.7	8.4
	0.5	0.558	112	5.9	0.504	90.3	6.6
	2	2.030	101	1.3	1.896	93.5	3.6
3,4-DCA	0.5	0.460	91.9	4.0	0.426	92.7	7.3
	2	1.850	92.7	3.0	1.680	90.6	4.2
	15	13.55	90.3	3.1	12.47	92.1	7.4
3,5-DCA	0.5	0.483	96.6	10.8	0.455	94.2	11.8
	2	1.955	97.8	2.0	1.838	94.0	5.4
	15	13.57	90.5	7.8	12.99	95.7	9.7

* Mean Conc. = Mean concentration

**Table 4:** Analysis of commercial rice samples.

Target Analytes	Mass Fraction (mg/kg)									
	S1	S2	S3	S4	S5	S6	S7	S8	S9	S10
DnBP	nd	0.15	nd	nd	0.11	nd	0.07	nd	nd	nd
BBP	0.11	nd	0.25	nd	nd	0.1	nd	0.05	0.18	0.12
DnOP	0.07	nd	nd	nd	0.08	nd	nd	0.1	nd	nd
DEHP	0.14	0.28	nd	nd	0.15	0.25	0.09	nd	0.14	nd
mBP	nd	nd	nd	nd	nd	nd	nd	nd	nd	nd

nd = not detectable; mass fraction below LOD.

## Data Availability

Not applicable.

## Supplementary Materials

The following supporting information can be downloaded at: https://www.mdpi.com/article/10.3390/foods11101482/s1, Figure S1. Matrix effect (%) of target analytes, in one concentration in un-diluted (dilution factor; df = 1) and diluted samples (df = 2, 3 and 4).

## References

[1] Liu L., Waters D.L.E., Rose T.J., Bao J., King G.J. (2013). Phospholipids in Rice: Significance in Grain Quality and Health Benefits: A Review. Food Chem..

[2] Jardim A.N.O., Caldas E.D. (2012). Brazilian Monitoring Programs for Pesticide Residues in Food—Results from 2001 to 2010. Food Control.

[3] Tsochatzis E.D., Tzimou-Tsitouridou R., Menkissoglu-Spiroudi U., Karpouzas D.G., Katsantonis D. (2013). Laboratory and Field Dissipation of Penoxsulam, Tricyclazole and Profoxydim in Rice Paddy Systems. Chemosphere.

[4] Tsochatzis E.D., Menkissoglu-Spiroudi U., Karpouzas D.G., Tzimou-Tsitouridou R. (2010). A Multi-Residue Method for Pesticide Residue Analysis in Rice Grains Using Matrix Solid-Phase Dispersion Extraction and High-Performance Liquid Chromatography–Diode Array Detection. Anal. Bioanal. Chem..

[5] Tsochatzis E.D., Tzimou-Tsitouridou R., Menkissoglu-Spiroudi U., Karpouzas D.G., Papageorgiou M. (2012). Development and Validation of an HPLC-DAD Method for the Simultaneous Determination of Most Common Rice Pesticides in Paddy Water Systems. Int. J. Environ. Anal. Chem..

[6] Lindh C.H., Littorin M., Amilon Å., Jönsson B.A.G. (2007). Analysis of 3,5-Dichloroaniline as a Biomarker of Vinclozolin and Iprodione in Human Urine Using Liquid Chromatography/Triple Quadrupole Mass Spectrometry. Rapid Commun. Mass Spectrom..

[7] González-Sálamo J., Socas-Rodríguez B., Hernández-Borges J. (2018). Analytical Methods for the Determination of Phthalates in Food. Curr. Opin. Food Sci..

[8] Simoneau C., Hannaert P. (1999). Stability Testing of Selected Plastics Additives for Food Contact in EU Aqueous, Fatty and Alternative Simulants. Food Addit. Contam..

[9] Tsochatzis E.D., Tzimou-Tsitouridou R., Gika H.G. (2017). Analytical Methodologies for the Assessment of Phthalate Exposure in Humans. Crit. Rev. Anal. Chem..

[10] Giuliani A., Zuccarini M., Cichelli A., Khan H., Reale M. (2020). Critical Review on the Presence of Phthalates in Food and Evidence of Their Biological Impact. Int. J. Env. Res. Public Health.

[11] Wenzl T., European Commission, Joint Research Centre, Institute for Reference Materials and Measurements (2009). Methods for the Determination of Phthalates in Food: Outcome of a Survey Conducted among European Food Control Laboratories.

[12] Kamrin M.A. (2009). Phthalate Risks, Phthalate Regulation, and Public Health: A Review. J. Toxicol. Environ. Health Part B.

[13] Silva M., Samandar E., Preaujr J., Reidy J., Needham L., Calafat A. (2007). Quantification of 22 Phthalate Metabolites in Human Urine. J. Chromatogr. B.

[14] Guo Y., Kannan K. (2012). Challenges Encountered in the Analysis of Phthalate Esters in Foodstuffs and Other Biological Matrices. Anal. Bioanal. Chem..

[15] Diamantidou D., Begou O., Theodoridis G., Gika H., Tsochatzis E., Kalogiannis S., Kataiftsi N., Soufleros E., Zotou A. (2019). Development and Validation of an Ultra High Performance Liquid Chromatography-Tandem Mass Spectrometry Method for the Determination of Phthalate Esters in Greek Grape Marc Spirits. J. Chromatogr. A.

[16] US Environmental Protection Agency (2012). Phthalates: Action Plan 2012.

[17] Net S., Semper R. (2015). Occurrence, Fate, Behavior and Ecotoxicological State of Phthalates in Different Environmental Matrices. Environ. Sci. Technol..

[18] Gallart-Ayala H. (2013). Recent Advances in LC-MS Analysis of Food-Packaging Contaminants. Trends Anal. Chem..

[19] González-Sálamo J., Hernández-Borges J., Afonso M.D.M., Rodríguez-Delgado M.Á. (2018). Determination of Phthalates in Beverages Using Multiwalled Carbon Nanotubes Dispersive Solid-Phase Extraction before HPLC-MS. J. Sep. Sci..

[20] Silano V., Barat Baviera J.M., Bolognesi C., Chesson A., Cocconcelli P.S., Crebelli R., Gott D.M., Grob K., Lampi E., EFSA Panel on Food Contact Materials, Enzymes and Processing Aids (CEP) (2019). Update of the Risk Assessment of Di-butylphthalate (DBP), Butyl-benzyl-phthalate (BBP), Bis(2-ethylhexyl)Phthalate (DEHP), Di-isononylphthalate (DINP) and Di-isodecylphthalate (DIDP) for Use in Food Contact Materials. EFSA J..

[21] Pan Y.-L., Chen F., Zhang M.-Y., Wang T.-Q., Xu Z.-C., Zhang W., Chu Q.-C., Ye J.-N. (2013). Sensitive Determination of Chloroanilines in Water Samples by Hollow Fiber-Based Liquid-Phase Microextraction Prior to Capillary Electrophoresis with Amperometric Detection: CE and CEC. Electrophoresis.

[22] Sun H., Jiang F., Chen L., Zheng J., Wu Y., Liu M. (2014). Determination of Three Phthalate Esters in Environmental Samples by Coal Cinder Extraction and Cyclodextrin Modified Micellar Electrokinetic Chromatography. J. Chromatogr. Sci..

[23] Tsochatzis E., Karayannakidis P., Kalogiannis S. (2019). Determination of Selected Dichloroanilines and Phthalates in Lyophilised Mussels Samples with Ultra-High Performance Liquid Chromatography-Tandem Mass Spectrometry after QuEChERS Clean-Up. Food Addit. Contam. Part A Chem. Anal. Control Expo. Risk Assess..

[24] Tsochatzis E.D., Alberto Lopes J., Hoekstra E., Emons H. (2020). Development and Validation of a Multi-Analyte GC-MS Method for the Determination of 84 Substances from Plastic Food Contact Materials. Anal. Bioanal. Chem..

[25] Tsochatzis E.D., Lopes J.A., Gika H., Dalsgaard T.K., Theodoridis G. (2021). A Fast SALLE GC–MS/MS Multi-Analyte Method for the Determination of 75 Food Packaging Substances in Food Simulants. Food Chem..

[26] Guo Z., Wang S., Wei D., Wang M., Zhang H., Gai P., Duan J. (2010). Development and Application of a Method for Analysis of Phthalates in Ham Sausages by Solid-Phase Extraction and Gas Chromatography–Mass Spectrometry. Meat Sci..

[27] Anastassiades M., Lehotay S.J., Štajnbaher D., Schenck F.J. (2003). Fast and Easy Multiresidue Method Employing Acetonitrile Extraction/Partitioning and “Dispersive Solid-Phase Extraction” for the Determination of Pesticide Residues in Produce. J. AOAC Int..

[28] Farooq S., Wu H., Nie J., Ahmad S., Muhammad I., Zeeshan M., Khan R., Asim M. (2022). Application, Advancement and Green Aspects of Magnetic Molecularly Imprinted Polymers in Pesticide Residue Detection. Sci. Total Environ..

[29] Farooq S., Nie J., Cheng Y., Bacha S.A.S., Chang W. (2020). Selective Extraction of Fungicide Carbendazim in Fruits Using Β-cyclodextrin Based Molecularly Imprinted Polymers. J. Sep. Sci..

[30] Li Y., Nie J., Chang W., Xu G., Farooq S., Liu M., Zhang J. (2020). Enantioselective Behavior Analysis of Chiral Fungicide Tetraconazole in Apples with UPLC-MS/MS. Food Control.

[31] Kataoka H., Ise M., Narimatsu S. (2002). Automated On-Line in-Tube Solid-Phase Microextraction Coupled with High Performance Liquid Chromatography for the Analysis of Bisphenol A, Alkylphenols, and Phthalate Esters in Foods Contacted with Plastics. J. Sep. Sci..

[32] Sannino A. (2010). Development of a Gas Chromatographic/Mass Spectrometric Method for Determination of Phthalates in Oily Foods. J. AOAC Int..

[33] Perestrelo R., Silva C.L., Algarra M., Câmara J.S. (2021). Evaluation of the Occurrence of Phthalates in Plastic Materials Used in Food Packaging. Appl. Sci..

[34] Guo D., Yi X., Qu L. (2011). Determination of linuron and its metabolite 3,4-dichloroaniline residues in meat and meat products using liquid chromatography-tandem mass spectrometry. Se Pu.

[35] Meghesan-Breja A., Cimpoiu C., Hosu A. (2017). Identification and Quantification of Some Pesticide Metabolites from Vegetables by GC-TOF-MS and LC-MS-QQQ. Studia Univ. Babes-Bolyai Chem..

[36] Cordeiro F., Karasek L., Cizek-Stroh A., Charoud-Got J., Robouch P., Hoekstra E. (2019). Determination of Phthalates in Food Simulant a Solution.

[37] (2011). European Commission Commission Regulation (EU) No 10/2011 on Plastic Materials and Articles Intended to Come into Contact with Food. https://eur-lex.europa.eu/legal-content/EN/TXT/PDF/?uri=CELEX:32011R0010&from=EN.

[38] European Commission, Directorate General for Health and Food Safety Guidance Document on Analytical Quality Control and Method Validation Procedures for Pesticide Residues and Analysis in Food and Feed (2017). SANTE/11813/2017. https://www.eurl-pesticides.eu/userfiles/file/EurlALL/SANTE_11813_2017-fin.pdf.

[39] Food and Drug Administration (FDA) Guidance for Industry—Q2B Validation of Analytical Procedures: Methodology 1997. https://www.gmp-compliance.org/files/guidemgr/1-12-5.pdf.

[40] Matuszewski B.K., Constanzer M.L., Chavez-Eng C.M. (2003). Strategies for the Assessment of Matrix Effect in Quantitative Bioanalytical Methods Based on HPLC−MS/MS. Anal. Chem..

[41] Tsochatzis E.D., Alberto Lopes J., Kappenstein O., Tietz T., Hoekstra E.J. (2020). Quantification of PET Cyclic and Linear Oligomers in Teabags by a Validated LC-MS Method—In Silico Toxicity Assessment and Consumer’s Exposure. Food Chem..

[42] Kruve A., Leito I., Herodes K. (2009). Combating Matrix Effects in LC/ESI/MS: The Extrapolative Dilution Approach. Anal. Chim. Acta.

[43] Tsochatzis E.D., Mieth A., Alberto Lopes J., Simoneau C. (2020). A Salting-out Liquid-Liquid Extraction (SALLE) for the Analysis of Caprolactam and 2,4-Di-Tert Butyl Phenol in Water and Food Simulants. Study of the Salinity Effect to Specific Migration from Food Contact Materials. J. Chromatogr. B.

[44] Yadav S., Rai S., Srivastava A.K., Panchal S., Patel D.K., Sharma V.P., Jain S., Srivastava L.P. (2017). Determination of Pesticide and Phthalate Residues in Tea by QuEChERS Method and Their Fate in Processing. Environ. Sci. Pollut. Res..

[45] Cao X.-L., Zhao W., Dabeka R. (2015). Di-(2-Ethylhexyl) Adipate and 20 Phthalates in Composite Food Samples from the 2013 Canadian Total Diet Study. Food Addit. Contam. Part A.

[46] US Environmental Protection Agency (US EPA) EPA, Method 8061A-Phthalate Esters by Gas Chromatography with Electron Capture Detection (GC/ECD). https://www.epa.gov/sites/default/files/2015-12/documents/8061a.pdf.

[47] Milan M., Vidotto F., Piano S., Negre M., Ferrero A. (2012). Dissipation of Propanil and 3,4 Dichloroaniline in Three Different Rice Management Systems. J. Environ. Qual..

[48] Škrbić B.D., Ji Y., Živančev J.R., Jovanović G.G., Jie Z. (2017). Mycotoxins, Trace Elements, and Phthalates in Marketed Rice of Different Origin and Exposure Assessment. Food Addit. Contam. Part B.

