# Supplementary material for: Development, Validation and Application of an Ultra-High-Performance Liquid Chromatography–Tandem Mass Spectrometry (UHPLC-MS/MS) Method after QuEChERS Cleanup for Selected Dichloroanilines and Phthalates in Rice Samples
Source: Foods. 2022 May 19;11(10):1482. doi: 10.3390/foods11101482 (PMC9140621; doi:10.3390/foods11101482)
Supplement: Supplementary file 1 [file foods-11-01482-s001.zip › foods-1738670-supplementary-done.pdf]

foods

MDPI

Supplementary material

Development, validation and application of an ultra-high performance liquid chromatography-tandem mass spectrometry (UHPLC-MS/MS) method after QuEChERS clean up for selected dichloroanilines and phthalates in rice samples

Emmanouil Tsochatzis [1,*], Olga Begou[2,3], Stavros Kalogiannis[3,4], Helen Gika[3,5], Emel Oz[1,6], Fatih Oz[1,6], Georgios Theodoridis[2,3]

[1] *Aarhus University, Department of Food Science, Agro Food Park 48, Aarhus 8200 N, Denmark; emmanouil.tsochatzis@food.au.dk (E.T.)*

[2] *Laboratory of Analytical Chemistry, Department of Chemistry, Aristotle University of Thessaloniki, University Campus, 54124 Thessaloniki, Greece; mpegolga@chem.auth.gr (O.B.); gtheodor@chem.auth.gr(G.T.)*

[3] *Biomic_AUTh, Center for Interdisciplinary Research and Innovation (CIRI-AUTH), Balkan Center, B1.4, 10th km Thessaloniki-Thermi Rd, P.O. Box 8318, 57001 Thessaloniki, Greece; Kalogian@ihu.gr (S.K.)*

[4] *International Hellenic University, Department of Nutritional Sciences and Dietetics, P.O. 141 Sindos, 57400, Thessaloniki, Greece*

[5] *Laboratory of Forensic Medicine and Toxicology, School of Medicine, Aristotle University of Thessaloniki, University Campus, 54124 Thessaloniki, Greece; gkikae@auth.gr*

[6] *Ataturk University, Agriculture Faculty, Food Engineering Department, 25240, Erzurum, Turkey; emel.oz@atauni.edu.tr (E.O.) fatihoz@atauni.edu.tr (F.O.)*

* *Correspondence: Emmanouil Tsochatzis (E.T.); emmanouil.tsochatzis@food.au.dk; Tel.: +306977441091 (Current Affiliation: European Food Safety Authority-EFSA, Via Carlo Magno 1A, 43146, Parma, Italy)*

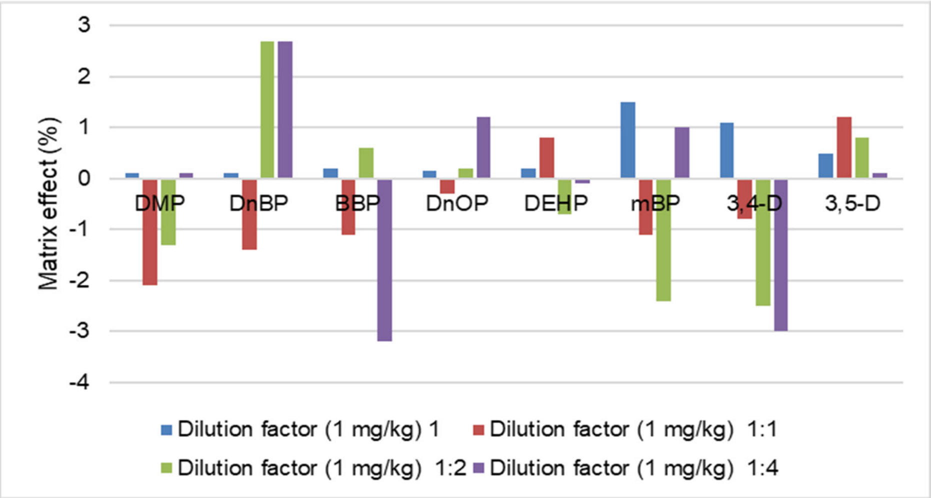

Figure S1. Matrix effect (%) of target analytes, in one concentration in un-diluted (dilution factor; df=1) and diluted samples (df=2,3 and 4).
